# Supplementary material for: Damage-responsive, maturity-silenced enhancers regulate multiple genes that direct regeneration in Drosophila
Source: eLife. 2020 Jun 3;9:e58305. doi: 10.7554/eLife.58305 (PMC7299344; doi:10.7554/eLife.58305)
Supplement: Transparent reporting form [file elife-58305-transrepform.docx]

***eLife’s* transparent reporting form**

We encourage authors to provide detailed information *within their submission* to facilitate the interpretation and replication of experiments. Authors can upload supporting documentation to indicate the use of appropriate reporting guidelines for health-related research (see [EQUATOR Network](http://www.equator-network.org/%20)), life science research (see the [BioSharing Information Resource](https://biosharing.org/)), or the [ARRIVE guidelines](http://www.plosbiology.org/article/info:doi/10.1371/journal.pbio.1000412) for reporting work involving animal research. Where applicable, authors should refer to any relevant reporting standards documents in this form.

If you have any questions, please consult our Journal Policies and/or contact us: [editorial@elifesciences.org](mailto:editorial@elifesciences.org).

**Sample-size estimation**

- You should state whether an appropriate sample size was computed when the study was being designed
- You should state the statistical method of sample size computation and any required assumptions
- If no explicit power analysis was used, you should describe how you decided what sample (replicate) size (number) to use

Please outline where this information can be found within the submission (e.g., sections or figure legends), or explain why this information doesn’t apply to your submission:

Sample sizes for wing scoring experiments were determined based on (1) previous experiments using the same or similar ablation and regeneration scoring approach (Smith-Bolton et al 2009, Harris et al. 2016), and (2) by comparing experiments conducted on control flies across multiple investigations (large sample size) to individual experiments (smaller sample sizes). From these and previous investigations it was determined that a sample size of at least 100 individual organisms ablated and scored for regeneration could be used to detect statistical significance reliably, and that larger sample sizes do not alter experimental conclusions. Total sample size used in each wing scoring experiments is given on the stacked bar graphs in each figure.

**Replicates**

- You should report how often each experiment was performed
- You should include a definition of biological versus technical replication
- The data obtained should be provided and sufficient information should be provided to indicate the number of independent biological and/or technical replicates
- If you encountered any outliers, you should describe how these were handled
- Criteria for exclusion/inclusion of data should be clearly stated
- High-throughput sequence data should be uploaded before submission, with a private link for reviewers provided (these are available from both GEO and ArrayExpress)

Please outline where this information can be found within the submission (e.g., sections or figure legends), or explain why this information doesn’t apply to your submission:

Each wing scoring experiment was performed using 6-12 separate vials of ~25 scorable larvae (technical replicates), while the entire experiment was then repeated at least once (biological replicate). Ablation and immunohistochemistry analysis of discs was performed with at least 3 pooled technical replicates and at least 2 biological replicates. Both protocols are outlined in the materials and methods section of the manuscript. ATAC-seq analysis was performed in triplicate (biological repeats from separate ablation experiments), as indicated in the outline accompanying the GEO database submission.

**Statistical reporting**

- Statistical analysis methods should be described and justified
- Raw data should be presented in figures whenever informative to do so (typically when N per group is less than 10)
- For each experiment, you should identify the statistical tests used, exact values of N, definitions of center, methods of multiple test correction, and dispersion and precision measures (e.g., mean, median, SD, SEM, confidence intervals; and, for the major substantive results, a measure of effect size (e.g., Pearson's r, Cohen's d)
- Report exact p-values wherever possible alongside the summary statistics and 95% confidence intervals. These should be reported for all key questions and not only when the p-value is less than 0.05.

Please outline where this information can be found within the submission (e.g., sections or figure legends), or explain why this information doesn’t apply to your submission:

Wing scoring experiments were analyzed using Fisher Exact test to measure the significance of difference from control samples ablated in parallel. Raw number of flies binned into each category (full, nicked, partial notch, full notch or ablated) were summed across technical replicates for a biological replicate and used to perform Fisher Exact in a 2x5 contingency table with the control distribution used as the expected values for each experiment. P-values are given in figure legends when significant (p<0.01), or as p<0.0001. Graphed wing scoring data is alternatively presented in Supplemental File 5, diagramed as individual bar charts with error bars instead of stacked bar charts. Error bars were generated using the proportion of flies in each category per technical replicate to calculate standard error for each category per experiment. ATAC-seq data were analyzed as indicated in the materials and methods section of the manuscript, and the outline accompanying the GEO database submission. Both unadjusted and adjusted p-values are provided in supplemental tables. Statistical cutoffs are given in the supplemental file legends, main text and materials and methods sections of the manuscript.

(For large datasets, or papers with a very large number of statistical tests, you may upload a single table file with tests, Ns, etc., with reference to sections in the manuscript.)

**Group allocation**

- Indicate how samples were allocated into experimental groups (in the case of clinical studies, please specify allocation to treatment method); if randomization was used, please also state if restricted randomization was applied
- Indicate if masking was used during group allocation, data collection and/or data analysis

Please outline where this information can be found within the submission (e.g., sections or figure legends), or explain why this information doesn’t apply to your submission:

Virgin flies used for ablation (ablation genotypes of DUAL Control or A3) were collected over several days, randomized and crossed to newly enclosed males of the required genotype without selection. For wing scoring, each experiment was scored by a single scientist using blinded genotypes.

**Additional data files (“source data”)**

- We encourage you to upload relevant additional data files, such as numerical data that are represented as a graph in a figure, or as a summary table
- Where provided, these should be in the most useful format, and they can be uploaded as “Source data” files linked to a main figure or table
- Include model definition files including the full list of parameters used
- Include code used for data analysis (e.g., R, MatLab)
- Avoid stating that data files are “available upon request”

Please indicate the figures or tables for which source data files have been provided:

Attributes and identities of ATAC-seq peaks and associated genes presented as volcano plots, Venn diagrams and summary tables in the manuscript are provided in Supplemental File 1-3. Data for regeneration scoring is provided in Supplemental File 5.
